# Positively increased visceral adiposity index in hyperuricemia free of metabolic syndrome

**DOI:** 10.1186/s12944-018-0761-1

**Published:** 2018-05-07

**Authors:** Dongfeng Gu, Yanan Ding, Yunfeng Zhao, Shuzhai Miao, Qingshan Qu

**Affiliations:** 1 Department of Nephrology and Transplantation Center, The People’s Hospital of Zhengzhou, Affiliated with Southern Medical University, Zhengzhou, 450003 People’s Republic of China; 2 Division of Cardiovascular Medicine, The People’s Hospital of Zhengzhou, Affiliated with Southern Medical University, Zhengzhou, 450003 People’s Republic of China

**Keywords:** Visceral adiposity index, Hyperuricemia, Metabolic syndrome, Obesity

## Abstract

**Background:**

Visceral adiposity index (VAI) was closely associated with metabolic syndrome, however almost no research focused on VAI and hyperuricemia, therefore, this study was conducted to determine the relationship of VAI and hyperuricemia free of metabolic syndrome and estimate the power of VAI as predictor for hyperuricemia.

**Methods:**

A cross-sectional research coming from a health check-up program was conducted. All participants were divided into four groups according to VAI quartiles. A multivariate logistic analysis was used to analyze the relationship between the quartiles and hyperuricemia. A receiver operating characteristic (ROC) curve analysis was used to evaluate the accuracy of predictions for hyperuricemia.

**Results:**

VAI was independent risk factor of hyperuricemia. The ORs of which in the upper quartile were 3.077 (95%CI 1.78-5.293), *P* = 0.000, in model 1, after adjusting for age, systolic blood pressure, diastolic blood pressure, heart rate, fast plasma glucose, serum creatinine, triglyceride, total cholesterol, high density lipoprotein cholesterol, and low density lipoprotein cholesterol; and 3.041 (95CI 1.767-5.233), *P* = 0.000, in model 2, after adjusting for the above plus physical activity, diet, smoking habits, alcohol consumption, hypertension and diabetes history. The area under the ROC curve (AUC) value of VAI was 0.618 (95%CI 0.572-0.665), *P* = 0.000; it was higher than WC, which was 0.556 (95%CI 0.508-0.604), *P* = 0.024, for hyperuricemia.

**Conclusions:**

VAI was associated with hyperuricemia among individuals free of metabolic syndrome, and also a powerful indicator.

## Background

There are two kinds of central obesity: subcutaneous and visceral fat mass, and waist circumference (WC) alone does not help distinguishing them [1]. The Visceral Adiposity Index (VAI), which is based on WC, body mass index (BMI), triglycerides(TG), and high density lipoprotein cholesterol (HDL-C) and was recently introduced by Amato et al. [2], was used as a marker of visceral fat dysfunction, and been suggested as an indicator of Metabolic syndrome (MetS) [3, 4]. MetS, which was defined as a distinct entity which comprises the following components: central obesity, dyslipidemia, hyperglycemia and hypertension, increase a person’s risk of cardiovascular disease [5, 6], and more and more studies revealed that hyperuricemia is a risk factor of MetS [7–9].

Serum uric acid is a clinically useful nutritional marker [10], and hyperuricemia has been a major health problem in the world; the prevalence of which was 9.9% in men (cutoff point = 7.0 mg/dl) and 7.0% in women (cutoff point = 6.0 mg/dl) according to a national cross-sectional survey in China [11], however the prevalence of serum urate levels > 6 mg/dl was 50.4% among men and 16.3% among women in US [12]. Asymptomatic hyperuricemia, may progress into gout which characterized as monosodium urate crystal deposition, and also was independently associated with prevalent hypertension [13]. Therefore, it has been a public health burden, especially in males.

However, there was almost no research on the relationship of VAI and hyperuricemia, and MetS may be a confounder, therefore, we initiated to do this research about VAI and hyperuricemia free of MetS in Chinses males, in order to study the relationship of VAI and hyperuricemia free of metabolic syndrome and estimate the power of VAI as predictor for hyperuricemia.

## Methods

### Participants

A cross-sectional research from 3 Jan, 2016 to 3 Jan, 2017 was conducted. Sample size was determined by statistician, and which was more than 288 at a given confidence level of 5%, confidence interval of 95%, and central obesity epidemiology of 24.9% in China referred from Lancet [14]. The Ethics Committee of The People’s Hospital of Zhengzhou approved the study. A total of 713 male adult participants were recruited from a self-paid health check-up program at the Health Management Centre of The People’s Hospital of Zhengzhou with individual informed consent, written or oral, according to the principles expressed in the Declaration of Helsinki. The information of their anthropometric measurements, biochemical data, medical history and medication usage were well documented; subjects with acute illness, malignancy, infection including of chronic hepatitis B and chronic hepatitis C, current users of anti-thyroid, glucocorticoids, unclear cold medicine, and non-steroid anti-inflammatory drugs, were excluded, especially MetS. Finally, 633 non-MetS male participants were included in our analysis.

### Questionnaire

Anthropometric measurements, medical history and medication usage were included in questionnaires as described in detail previously [15]. Participants were asked standing erect and had relaxed the abdominal muscles before WC measurement by a flexible inch tape. Measurement was taken at the end of normal expiration. Locate the top of the hip bone (iliac crest) and take the measurement just above this bony landmark, just where one finger can fit between the iliac crest and the lowest rib. The questionnaire consisted of questions regarding age, sex, a personal history of diabetes (yes vs. no), a personal history of hypertension (yes vs. no), a personal history of cardiovascular disease (yes vs. no), diet (balanced diet vs. unbalanced diet) according to Chinese Dietary Balance Index and Diet Quality Distance recommended by the Chinese Nutrition Society, Diet Quality Distance (range 0 to 84) was calculated based on DBI-07, 0-17 were looked as balanced diet, and 17-84 were looked as unbalanced diet [16], Physical activity (> 60 min/day vs. 30–60 min/day vs. < 30 min/day vs. no), > 60 min/day was considered as active physical activity, and < 30 min/day and no were considered as inactive physical activity based on our previous publication [15], smoking habits (yes[current] vs. no), alcohol intake (yes[current] vs. no); WC, height and systolic blood pressure (SBP) and diastolic blood pressure (DBP) were each measured manually [15]. Prior to BP measurements, participants were seated quietly for 5 to 10 min in a chair with arm supported at heart level and the rotator cuff positioned 3 cm above the antecubital fossa, BP was measured using Omron (SEM 1 Model) automatic BP monitor (Omron Healthcare Co., Ltd., IL, USA) with an appropriate cuff size [17, 18]. Heart rate (HR) was also documented from the automatic BP monitor. Average BP and HR were then calculated from three measurements. BMIs were calculated using the following equation: BMI = weight (kg)/height^2^ (m^2^).

### Biochemical data

Biochemical data acquiring Protocols was also described in detail in our previous publication [15]. Appointments were scheduled for blood collection. Fasting venous blood draws were performed at The People’s Hospital of Zhengzhou. All blood samples were sent to the central laboratory of The People’s Hospital of Zhengzhou. The blood samples were either disposed of within 3 h or stored at 4 °C for as long as 2 days. FPG (fasting plasma glucose, FPG) testing was performed via an electrochemical luminescence immunoassay. Serum total cholesterol (TC), HDLC, TG, and low-density lipoprotein cholesterol (LDLC) were each measured using an autoanalyzer (Toshiba, Japan). Serum creatinine (Scr) was measured using overnight fasting venous blood samples, via Jaffe’s kinetic method. Uric acid was detected by uricase-based spectrophotometry.

### Evaluation criteria

VAI, a sex-specific index based on WC, BMI, TG and HDLC, was calculated as follows [2]:

$$ \mathrm{Males}:\mathrm{VAI}=\left(\frac{\mathrm{WC}}{39.68+\left(1.88\times BMI\right)}\right)\times \left(\frac{\mathrm{TG}}{1.03}\right)\times \left(\frac{1.31}{\mathrm{HDL}}\right) $$

$$ \mathrm{Females}:\mathrm{VAI}=\left(\frac{\mathrm{WC}}{36.58+\left(1.89\times \mathrm{BMI}\right)}\right)\times \left(\frac{\mathrm{TG}}{0.81}\right)\times \left(\frac{1.52}{\mathrm{HDL}}\right) $$

MetS was defined as a composition of any 3 of 5 risk factors including of central obesity, raised triglycerides, lowered high-density lipoprotein cholesterol, raised fasting glucose and raised blood pressure [5, 6]. Hyperuricemia was defined as a serum uric acid (SUA) concentration exceeding the saturation point of 6.8 mg/dL (404 μmol/L) at physiologic pH and body temperature, without symptoms from crystal deposition [19].

### Statistical analysis

Acquired data were analyzed using SPSS 16.0 (SPSS Inc., Chicago, IL, USA). Continuous variables were represented as means ± SDs, and categorical variables were represented as proportions of each group. The basic characteristics of the four VAI quartiles were examined in non-MetS males. Continuous variables were analyzed via one-way ANOVA, and categorical variables were analyzed via the Chi-square test or the Fisher’s exact test.

Logistic regression models were used to determine whether VAI is associated with hyperuricemia free of MetS males. VAI was divided into four quartiles and considered a categorical variable. Model one was adjusted for age, VAI, SBP, DBP, heart rate, FPG, sCr, TC, TG, HDLC and LDLC; model two was adjusted for the above confounders, plus physical activity, diet, smoking habits, alcohol consumption, HP and DM. The lower quartile was used as a reference category. And logistic regression was also performed in analyzing the risk factor of VAI. *P* values less than 0.05 were considered statistically significant.

A receiver operating characteristic (ROC) curve analysis was used to evaluate the accuracy of predictions for hyperuricemia free of MetS males. The accuracy was showed as the area under the ROC curve (AUC) with 95% confidence interval (CI). To determine the appropriate cut-off point for each adiposity index, the score with the highest Youden’s index (sensitivity+specificity-1, Youden’s Index) was considered as the optimal cut-off one.

## Results

### The basic characteristics of the male and female participants

As shown in Table 1, there were significant differences in inactive physical activity, diabetes mellitus, WC, BMI, DBP, HR, SUC, serum glucose, TG, HDLC, TC, and LDLC in non-MetS males, as the upper VAI quartile participants exhibited higher DBP, serum glucose, TG, TC, and LDLC, prevalence of inactive physical activity, and lower HDLC, compared with the lower VAI subjects in non-MetS males.

**Table 1:** Baseline characteristics of Non-metabolic syndrome health check-up population

	VAI (male)				*P*
	1st Quartile (*n* = 162)	2nd Quartile (*n* = 162)	3rd Quartile (*n* = 151)	4th Quartile (*n* = 158)	
Age (years)	53.3 ± 16.6	54.4 ± 15.0	51.8 ± 13.8	52.7 ± 13.5	0.458
Inactive Physical Activity (%)	93(57.4%)	96(59.3%)	102(67.5%)	117(74.1%)	*P* < 0.05
Unbalanced Diet (%)	39(24.1%)	38(23.5%)	39(25.8%)	46(29.1%)	0.655
Current Smoking (%)	47(29.0%)	55(34.0%)	38(25.2%)	63(39.9%)	0.331
Current Alcohol (%)	25(15.4%)	21(13.0%)	22(14.6%)	26(16.5%)	0.842
Hypertension (%)	33(20.4%)	37(22.8%)	37(24.5%)	50(31.6%)	0.111
Diabetes Mellitus (%)	74(4.3%)	6(3.7%)	17(11.3%)	16(10.1%)	*P* < 0.05
Waist Circumference (cm)	79.7 ± 8.6	85.8 ± 8.7	90.0 ± 8.1	91.3 ± 7.9	*P* < 0.001
Body Mass Index (kg/m^2^)	21.9 ± 2.8	23.4 ± 3.0	24.6 ± 3.2	25.1 ± 2.7	*P* < 0.001
Systolic Blood Pressure (mmHg)	128.0 ± 20.4	129.8 ± 18.3	129.7 ± 17.0	132.3 ± 18.2	0.227
Diastolic Blood Pressure (mmHg)	76.5 ± 11.1	78.0 ± 10.2	80.6 ± 9.9	82.2 ± 9.6	*P* < 0.001
Heart rate (/min)	73.7 ± 11.3	72.6 ± 9.8	74.5 ± 9.6	76.8 ± 11.0	*P* < 0.05
Serum Creatinine (μmol/L)	85.5 ± 14.2	85.4 ± 15.3	88.7 ± 15.1	89.0 ± 16.4	0.051
Serum uric acid (mmol/L)	370.9 ± 83.0	385.4 ± 82.9	402.0 ± 83.9	431.0 ± 89.3	*P* < 0.001
Serum glucose (mmol/L)	4.8 ± 1.1	5.0 ± 1.2	5.2 ± 1.2	5.4 ± 1.6	*P* < 0.05
Triacylglycerols(mmol/L)	0.8 ± 0.2	1.1 ± 0.2	1.8 ± 0.4	3.4 ± 1.6	*P* < 0.001
Total Cholesterol (mmol/L)	5.1 ± 1.0	5.3 ± 0.9	5.5 ± 1.0	5.5 ± 0.9	*P* < 0.001
High-Density Lipoprotein Cholesterol (mmol/L)	1.7 ± 0.3	1.4 ± 0.2	1.3 ± 0.2	1.3 ± 0.3	*P* < 0.001
Low-Density Lipoprotein Cholesterol (mmol/L)	3.0 ± 0.8	3.4 ± 0.9	3.3 ± 0.9	3.9 ± 0.9	*P* < 0.01

Means ± SDs represented the continuous variables, and proportions represented the categorical variables

1st quartile of VAI: 0-0.78; 2nd quartile of VAI: 0.79-1.18; 3rd quartile of VAI: 1.19-1.90; 4th quartile of VAI: ≥1.90

Continuous variables were analyzed via One-way ANOVA, categorical variables were analyzed via the Chi-square test or Fisher’s exact test, and *P* value less than 0.05 was considered statistical significant

### The relationship between VAI and hyperuricemia free of MetS males

As shown in Table 2, the VAI was significantly associated with hyperuricemia free of MetS males, after adjusting for age, SBP, DBP, HR, FPG, sCr, TG, TC, HDLC, and LDLC; the ORs for hyperuricemia in the upper quartile of the VAI were 3.077 (95%CI 1.78-5.293), *P* = 0.000, in non-MetS males. Following further adjustments for the above confounders, physical activity, diet, smoking habits, alcohol consumption, HP and DM, the ORs for hyperuricemia in the upper quartile of the VAI were 3.041 (95CI 1.767-5.233), *P* = 0.000, in non-MetS males.

**Table 2:** The relationship between visceral adiposity index and hyperuricemia free of metabolic syndrome males

VAI	Model one ^a^		Model two ^b^	
	OR (95% CI)	*P*	OR (95% CI)	*P*
1st Quartile	Reference		Reference	
2nd Quartile	1.513 (0.896-2.556)	0.121	1.514 (0.896-2.557)	0.121
3rd Quartile	2.101 (1.246-3.542)	0.005	2.102 (1.247-3.543)	0.005
4th Quartile	3.077 (1.789-5.293)	0.000	3.041 (1.767-5.233)	0.000

^a^ Adjusted for age, SBP, DBP, heart rate, FPG, sCr, triglyceride, total cholesterol, high density lipoprotein cholesterol, and low density lipoprotein cholesterol; ^b^ Adjusted for the above plus physical activity, diet, smoking habits, alcohol consumption, HP and DM

Table 3 summarized the ability of three indices to predict hyperuricemia free of MetS males. The AUC value of VAI was 0.618 (95%CI 0.572-0.665), *P* = 0.000, and the cut-off values of which was 1.15 for hyperuricemia. The AUC value of WC was 0.556 (95%CI 0.508-0.604), *P* = 0.024, and the cut-off values of which was 79.5 for hyperuricemia. BMI has no significant meaning, *P* = 0.810.

**Table 3:** The power of VAI, WC and BMI in hyperuricemia free of metabolic syndrome males

	AUC	(95% CI)	*P* value	Cut off	Sensitivity	1-Specificity	Yonden’s index
VAI	0.618	0.572-0.665	0.000	1.15	0.628	0.428	0.200
WC	0.556	0.508-0.604	0.024	79.5	0.781	0.655	0.126
BMI	0.494	0.445-0.543	0.810	19.05	0.939	0.901	0.038

**Fig. 1:**
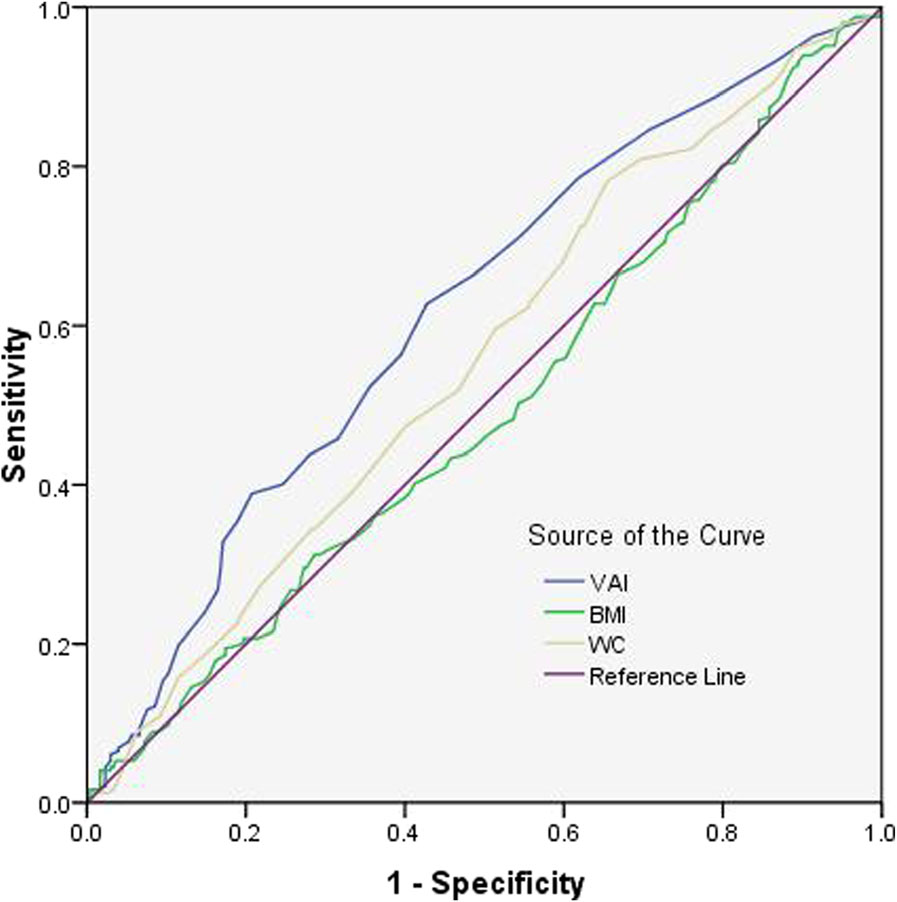
The discriminatory power of VAI, WC and BMI in asymptomatic hyperuricemia free of metabolic syndrome males

Figure 1 compared the accuracy of VAI, WC and BMI for predicting hyperuricemia free of MetS males. Both VAI and WC were positively correlated with hyperuricemia, and VAI had the highest AUC.

## Discussion

The study revealed that there was a significantly relationship between VAI and hyperuricemia free of MetS males. Risk of hyperuricemia was higher in the upper quartile of VAI compared to the lower quartile of VAI, the OR was 3.077 (1.789-5.293), *P* = 0.000. Our pilot study also performed a multivariate logistic regression using VAI values as continuous variables, which revealed that VAI was also independent risk factor of hyperuricemia free of MetS males, the OR was 2.811 (2.128-3.714), *P* = 0.000. VAI had the highest AUC compared with WC and BMI in hyperuricemia free of MetS males, which was not revealed in the literature to the best of our knowledge.

Some anthropometric indices of obesity had been studied to explore the relationship of which with hyperuricemia. Body shape index (BSI), body roundness index (BRI), BMI, WC and waist-to -height ratio (WHtR) were significantly associated with hyperuricemia in both males and females, and BRI rather than ABSI showed a superior predictive ability for identifying hyperuricemia than BMI in female and similar capabilities as those of WC and WHtR in the female, but not in the male gender [20]. BMI increase and hypertriglyceridemia may potentiate serum uric acid effect on gout development [21]. Another Chinese research found that WC was strongly correlated with hyperuricemia in the Asian Mongolian area [22]. One research revealed that metabolically healthy obese, which means obese individuals with a favorable metabolic profile, had a high risk for hyperuricemia among Chinese general population [23, 24]. All of these studies were not excluded of MetS, and it was still controversial which one of anthropometric indices was superior to others. However, our research reveals that VAI was superior to WC in predicting of hyperuricemia free of MetS.

VAI not only dramatically increased with SUA, but also significantly increased with DBP, and FPG, as shown in Table 1. This has been already verified in literatures. VAI was significantly increased in prehypertension and hypertension in our previous report [15]. Some studies also found that VAI was associated with diabetes [25–27]. However, the underlying mechanism is puzzling. SUA was characterized as both pro-oxidant and antioxidant properties, depending on the context, and it can impair endothelial function, result to atherosclerotic risk [28–30]. New founding was that Visceral fat, not subcutaneous depot, exhibited greater expression of proinflammatory, oxidative stress-related, hypoxia-induced, and proangiogenic genes; increased activated macrophage populations; and had a higher capacity for cytokine production ex vivo; thus providing clinical evidence that visceral microenvironment play key important role in atherosclerotic vascular disease [31]. Excess visceral adiposity induces alterations in mitochondrial function and energy metabolism in tumor [32], and correlated with the expression of genes related to inflammation and oxidative stress in peripheral blood cells [31]. Visceral fat adiposity was associated with sub-clinical inflammation and increased oxidative stress [33]. Therefore, the mechanism of VAI intermediated hyperuricemia was remained to be revealed.

Inactive physical activity has positively with VAI in our study. As is known dyslipidemia was the basic of VAI increase. One Mexican population study revealed that sweetened beverages intake increased the risk of hyperuricemia and obesity [34]. Maybe keep fit and diet control was helpful for visceral obesity, specially in Chinese males, since Chinese individuals had the most deleterious abdominal visceral fat distribution and accumulation other than Europeans at a given BMI or WC [35]. A prospective study should be conducted to identify their causal relationship. However, it’s time to control visceral obesity.

Interleukin-6 (IL-6), tumor necrosis factor-alpha (TNF-α), and high sensitive C-reactive protein (hs-CRP) were also measured in order to determine the relationship of indices of inflammation with VAI and hyperuricemia. Our research also revealed that IL-6, TNF-α, and hs-CRP levels are significantly increased with hyperuricemia (IL-6: *r* = 0.308, *P* < 0.05; TNF-α: *r* = 0.365, *P* < 0.05; hs-CRP: *r* = 0.420, *P* < 0.01), and also have positive correlation with VAI (IL-6: *r* = 0.348, *P* < 0.05; TNF-α: *r* = 0.405, *P* < 0.05; hs-CRP: *r* = 0.496, *P* < 0.01). It’ well known that adipocytes can synthesize TNF-α and IL-6, the synthesis of C-reactive protein (CRP) in hepatocytes is IL-6 dependent, and CRP is also associated with adipose tissue [36]. High-fat and/or calorie-rich diet may also enhance production of these proinflammatory cytokines [37]. VAI has been associated with an increase in IL-6, TNF-α, hs-CRP in obese patients [38]. TNF-α has effects on TG through adipose tissue and liver TG metabolic pathways by increasing the level of proinflammatory cytokines such as IL-6 [38, 39]. It well documented that serum uric acid was also positively associated with IL-6, TNF-α, and hs-CRP [40–42]. Therefore, adiposity may be an inflammatory condition. However, the underlying mechanism is still to be revealed in the future.

### Limitations

The VAI was established in Caucasian populations, its suitability for other populations needs to be further confirmed. It remains to develop a well-designed epidemiological study to determine the underling mechanism.

## Conclusions

VAI was independently associated with hyperuricemia among individuals free of MetS and also a powerful indicator.

## Abbreviations

AUC: Area under the ROC
BMI: Body mass index
BRI: Body roundness index
BSI: Body shape index
CI: Confidence interval
CRP: C-reactive protein
DBP: Diastolic blood pressure
DM: Diabetic mellitus
FPG: Fasting plasma glucose
HDL-C: High density lipoprotein cholesterol
HP: Hypertension
HR: Heart rate
hs-CRP: High sensitive C-reactive protein
IL-6: Interleukin-6
LDL-C: Low-density lipoprotein cholesterol
MetS: Metabolic syndrome
OR: Odds ratio
ROC: Receiver operating characteristic
SBP: Systolic blood pressure
Scr: Serum creatinine
SUA: Serum uric acid
TC: Total cholesterol
TG: Triglycerides
TNF-α: Tumor necrosis factor-alpha
VAI: Visceral adiposity index
VAT: Visceral adiposity
WC: Waist circumference
WHtR: Waist to height ratio
